# Antioxidant Properties of Protein-Rich Plant Foods in Gastrointestinal Digestion—Peanuts as Our Antioxidant Friend or Foe in Allergies

**DOI:** 10.3390/antiox12040886

**Published:** 2023-04-05

**Authors:** Ivana Prodić, Maja Krstić Ristivojević, Katarina Smiljanić

**Affiliations:** 1 Innovative Centre of the Faculty of Chemistry in Belgrade Ltd., University of Belgrade, Studentski Trg 12–16, 11158 Belgrade, Serbia; 2 Institute of Molecular Genetics and Genetic Engineering, University of Belgrade, Vojvode Stepe 444a, 11042 Belgrade, Serbia; 3 Centre of Excellence for Molecular Food Sciences, Department of Biochemistry, Faculty of Chemistry, University of Belgrade, Studentski Trg 12–16, 11158 Belgrade, Serbia

**Keywords:** antioxidant peptides, bioactive peptides, food allergens, food matrix, gastrointestinal digestion, non-digestible polysaccharides, lipids, peanut, polyphenolics

## Abstract

Thermally processed peanuts are ideal plant models for studying the relationship between allergenicity and antioxidant capacity of protein-rich foods, besides lipids, carbohydrates and phytochemicals. Peanut is highly praised in the human diet; however, it is rich in allergens (>75% of total proteins). One-third of peanut allergens belong to the products of genes responsible for the defence of plants against stress conditions. The proximate composition of major peanut macromolecules and polyphenols is reviewed, focusing on the identity and relative abundance of all peanut proteins derived from recent proteomic studies. The importance of thermal processing, gastrointestinal digestion (performed by INFOGEST protocol) and their influence on allergenicity and antioxidant properties of protein-rich plant food matrices is elaborated. Antioxidant properties of bioactive peptides from nuts were also considered. Moreover, there are no studies dealing simultaneously with the antioxidant and allergenic properties of protein- and polyphenol-rich foods, considering all the molecules that can significantly contribute to the antioxidant capacity during and after gastrointestinal digestion. In summary, proteins and carbohydrates are underappreciated sources of antioxidant power released during the gastrointestinal digestion of protein-rich plant foods, and it is crucial to decipher their antioxidant contribution in addition to polyphenols and vitamins before and after gastrointestinal digestion.

## 1. Introduction

The importance of endogenous and dietary antioxidants for maintaining human health seems undeniable, as they regulate the levels of reactive oxygen/nitrogen/chlorine species. Plant foods are inevitable for these processes [1] because, compared to foods of animal origin, they have much higher polyphenols, fibres and phytochemicals, a diverse reservoir of antioxidants in the diet. On the other hand, within the top 20 protein families associated with food allergies, plant foods have a more diverse allergen portfolio than foods of animal origin (13 different protein families versus only four), with one family shared and the other belonging exclusively to the kingdom of fungi [2]. Even when counting all established allergens, *Plantae* “outperforms” *Animalia* by at least 10% (2 February 2023, http://allergen.org/treeview.php).

The mixture of plant proteins, lipids, carotenoids, other phenolics, vitamins and carbohydrates creates extensive interactions between macro and small molecules (non-covalent and covalent interactions) to combat immunological disorders, allergies and ageing, which are absorbed by the gastrointestinal tract (GIT) [3]. In addition, the complexity of the plant food matrix extends to pro-oxidants such as iron, copper, lipid peroxides, aldehydes, certain phenolic compounds and some proteins such as lipoxygenases [3]. Therefore, the oxidative stability of foods is related to the balance between antioxidant and pro-oxidant factors.

Nuts and certain beans such as soybeans and peanuts are among the primary plant-based candidates for a whole food that has the necessary properties, such as the presence of all essential amino acids [4,5] and the abundance of other important macromolecules (e.g., lipids and carbohydrates, including fibres) besides other phytochemicals that are promoted as dietary antioxidants [6]. Moreover, while peanut is the complete food, containing both lipids and proteins in considerable amounts, it is possible to additionally increase the oxidative stability of incomplete foods by various manipulations. For example, by protecting endogenous antioxidant enzymes, increasing the activity of proteins naturally occurring in foods by altering their structure, like hydrolysate preparation [7], introducing antioxidant proteins by genetic engineering or adding proteins or peptides with antioxidant activity [8,9,10].

The main targets of oxidative reactions affecting food quality are lipids and proteins. In lipids, these reactions usually involve free radicals forming lipid hydroperoxides, which eventually decompose to low molecular weight carbonyls in pro-oxidants [11]. These carbonyls are associated with stale odour and taste and can interact with compounds such as proteins to change functionality [12]. However, free radical mechanisms are also involved in protein oxidation, where the oxidation of amino acid residues within the protein occurs, with particular emphasis on those amino acids containing sulphur, amino or aromatic groups in a side chain [13]. The ability of proteins to interact with free radicals in foods makes them an essential component of antioxidant defence [13] and is being used as new antioxidant tactics to protect unsaturated fatty acids from oxidation [14] since they are more susceptible. For example, hydrolysates from whey and soy inhibited lipid oxidation in foods such as pork patties [14].

The allergenic potential of food can decrease, remain unchanged or even increase as a result of food processing [15], presumably as can its antioxidant potential. Thermal treatments alter the antioxidative potential and bioaccessibility of food [15] undergoing digestion in GIT. Thermal processing can cause non-enzymatic glycation, creating antioxidant and/or pro-oxidant species in the Maillard reaction [16]. This, in turn, may increase antioxidative power, correlating with the intensity of thermal treatment and the extent of Maillard products formed, as in the case of spray-dried camel milk [17]. In addition, thermal processing may alter the protein profile, including the associated modification profile [18] and/or the amount of food molecules extracted into the liquid phase of gastrointestinal digestion, directly affecting allergenicity [19] and, very likely, altering antioxidant potential.

When studying the antioxidant properties of protein-rich plant foods, the focus is usually on phytochemicals such as polyphenols and vitamins rather than on proteins or carbohydrates. In addition, most studies focus on the antioxidant properties of foods or some of their constituents before they enter the GIT than on gastric or gastrointestinal digestion food products. Recently, some studies have been published to monitor antioxidant capacity during gastrointestinal digestion of raw and thermally processed complex plant foods rich in proteins [20,21]. However, there is no paralleled research assessing foods’ antioxidant and allergenic potential during digestion in such complete food matrices. Given the biological importance of proteins as antioxidants [13], this review aims to summarise the contribution of proteins in the antioxidative food potential, besides renowned plant polyphenolics.

Furthermore, this review introduces the premise that proteins, besides polysaccharides, are the underestimated source of antioxidative power when portrayed through the gastrointestinal process, with peanuts as a role model for complex protein-rich plant foods, with significant amounts of allergens. Namely, high levels of proteins/allergens can generate plenty of di- and tri-peptides and free amino acids during digestion that could act as antioxidants in nuts (peanuts, hazelnuts, walnuts) and play an essential role in protecting the GIT itself from oxidative damage. In addition, the interaction between small and macromolecules are taken into account. Finally, a special section is devoted to antioxidant peptides with additional biological functions released from plant foods (food-derived bioactive peptides). In summary, the review presents the importance of deciphering the actual antioxidant contribution of all significant molecular species in peanuts before and after gastric and gastrointestinal digestion, considering the proximal composition, bioaccessibility, antioxidant activity and immune properties of allergenic proteins/peptides obtained during digestion.

## 2. Peanut as a Functional, Plant-Based Food

Peanuts have a high lipid and protein content with the following nutritional composition: about 50% oil, 25% protein, 15% carbohydrates, 2% fibre, 2% ash and 6% water [22]. Nuts, including peanuts (30 g), officially classified as legumes, provide over 10% of adult men’s recommended dietary allowance for protein, iron, thiamine and vitamin E [23]. They are also much more affordable than other nuts [24]. According to the amino acid index corrected for protein digestibility, soy protein is nutritionally of better quality than peanut protein [25]. On the other hand, a comparison of the apparent and standardised ileal digestibility of peanut flour and full-fat soybeans showed that they were relatively greater for crude proteins and the majority of amino acids in peanut flour than for soybeans in the study conducted on pigs [26]. Peanuts contain a small amount of sugar but provide more energy than the same amount of soybeans and contain much more vitamins B3, B5 and E than soybeans (https://foodstruct.com/compare/peanut-vs-soybean). In addition, some natural antioxidant constituents such as dihydroquercetin, luteolin and ethyl protocatechuate are reportedly present in the kernel and shell of peanuts [27]. Furthermore, resveratrol, phenolic acids, flavonoids and phytosterols that block the absorption of cholesterol from the diet are also abundant in peanuts, including coenzyme Q10; therefore, peanuts are considered a promising functional food [24].

### 2.1. Peanut Proteins and Allergens as a Potential Reservoir of Antioxidant Power

Food sources, which contain all essential amino acids in the required proportions, are termed complete proteins, such as casein (milk) and albumin (egg). The nutritional value of proteins in any food is reflected by its protein content, amino acid composition and protein digestibility. Peanuts contain all the essential amino acids necessary for protein synthesis and can therefore be an indispensable part of the human diet [24]. The World Health Organisation (WHO) recommends an amount of 0.83 g/kg of body weight protein for a healthy adult person [28]. According to numerous studies, peanuts contain more protein (25–30% depending on the variety) than any other legume or nut, except the soybean, which contains 37–39% (1 February 2023, https://foodstruct.com/compare/peanut-vs-soybean).

In addition, peanuts are an affordable and vital source of protein in third-world countries [24]. Peanut proteins have been found to have good emulsifying activity, stability, foaming capacity, excellent water retention and high solubility [24] and can also provide a new high-protein product formulation in the food industry [29]. For example, the protein content can reach 50% after peanut oil extraction [30].

Although the amino acid composition of peanuts varies greatly depending on the variety and location of the plant, peanuts contain all 20 amino acids in variable proportions [24]. In addition, peanut protein is rich in asparagine/aspartic acid and glutamine/glutamic acid but poor in methionine, threonine and tryptophan [31]. Peanuts are the richest in arginine (~3.5 g/100 g of protein), among the other foods, and Arg is the precursor to nitric oxide, a secondary messenger that dilates blood vessels and thus can lower blood pressure and reduce the risk of heart disease [32]. Phenylalanine, an essential amino acid, is abundant in peanuts (~6.1 g/100 g of protein) and serves as a precursor for epinephrine, norepinephrine and dopamine biosynthesis [24].

Many antioxidant mechanisms of proteins depend on amino acid composition (e.g., metal chelation, free radical scavenging, hydroperoxide reduction and aldehyde adduction). However, the antioxidant activity of these proteins is limited by the tertiary structure of the polypeptide because many amino acids with antioxidant potential can be buried inside the protein core, where they are inaccessible to pro-oxidants. For example, in natural β-lactoglobulin obtained from cows’ milk, most of the free radical scavenging amino acids are located in the protein’s interior and, therefore, may not be able to contribute to the overall antioxidant activity of the protein [33]. Therefore, these amino acids are unlikely to have significant free radical scavenging activity when the protein is in its native state. One approach to increasing the overall antioxidant activity of proteins is to disrupt the tertiary structure (i.e., partial denaturation), which can potentially increase the solvent accessibility of oxidatively labile amino acid residues.

The most abundant peanut proteins are two globulin families, arachin and conarachin, representing approximately 95% of the total proteins [34]. The information in Table 1 represents a relative quantitative and qualitative summary and merge of several publicly available mass spectrometry identification data obtained from the mature seeds of the peanut species *Arachis hypogaea*. Table 1 was compiled to show the number and diversity of protein families identified in the mature peanut seeds, with each family represented by at least one non/redundant protein group. All allergenic proteins were manually checked by align and blast tools to remove redundancies in the UniProt database. Table 1 is comprised mainly of our data involving the relative quantification of peanut proteins based on the intensity from extracted ion chromatographic-XIC curve areas in the control and gastric phase of digestion under INFOGEST protocol [19,35]. In addition, our data from the neutral phosphate/buffered saline (PBS) extract [18] and identification data from Hebling et al., 2012 (without the relative abundance due to the different approach used—spectral counts) [36] are presented in Table 1, among other studies. There are 62 distinct families of peanut proteins spread into 140 different, non-redundant protein groups (Table 1), with more than 500 iso- and proteoforms, as determined by high-resolution 2D SDS-PAGE [37]. Table 1 can be used to estimate the change in antioxidant potential due to the release of peptides under the action of pepsin, trypsin and chymotrypsin, taking into account available data [18,19,36], in silico digestion scenarios and already known properties of small peptides conferring antioxidant properties.

If not otherwise noted, qualitative data are extracted from the Supplementary tables of Hebling et al., 2012 [36] (e.g., all empty, merged cells of relative abundance without a specific reference). Qualitative data are represented by only one dominant protein group per protein family and not by all detected proteins within a specific protein family.

#### Peanut Allergy and Allergens

Previous positive information on peanut protein content and diversity does not have the same implications for peanut-allergic individuals, as allergic reactions to peanuts generally do not decrease with age. Peanuts are the most common food worldwide to cause severe or fatal anaphylaxis, even in small amounts [41]. Allergy to peanuts and tree nuts is reported to affect 1.4% of adults and 2.1% of children under 18 years of age in the general population [42]. The number of deaths related to food allergies in the U.S. population is approximately 1–3 deaths per 10 million people per year [41].

By correlating sensitisation patterns with clinical features, it has become possible to distinguish proteins responsible for primary sensitisation (complete allergens) from those that are more likely cross-reactive [2]. While evolutionary distance seems to be an essential factor in determining allergenicity for animal allergens, this aspect in relation to humans seems irrelevant for plant allergens. Until we know more about the mechanistic details of primary sensitisation, including the involvement of molecules that stimulate innate immune responses and the repertoire of T-cell and B-cell antigens [43], we will not be able to explain fundamental questions, such as why peanut allergy is more severe than soy allergy [2], or what is the precise role of the increased antioxidant milieu in the GIT in a specific type of food allergy, e.g., peanut allergy.

Currently, 17 different peanut allergens are recognised officially and classified into ten different structural protein families, where the last newcomer is from the cyclophilin–peptidyl-prolyl cis-trans isomerase family (Ara h 18 allergen), based on the WHO/IUIS Allergen Nomenclature Subcommittee (18 January 2023, http://www.allergen.org) (Table 1). The major peanut allergens are Ara h 1, Ara h 2, Ara h 3, representing the most common causes of allergic reactions worldwide [42], including Ara h 6 (not officially counted among the major allergens), with a high structural homology to Ara h 2 and complement Ara h 2 as an important marker for IgE reactivity to peanut [44]. All the allergenic groups belong to minor peanut allergens (Ara h 7-Ara h 18). Lectin PNA belongs to the group of seed storage proteins as the major peanut allergens are designated as Ara h agglutinin and are considered a minor peanut allergen [42]; however, it is not officially recognised by the WHO/IUIS Committee.

The major allergens (Ara h 1–3) account for 75% of the total protein content, while all allergens account for up to 85% of the total protein content of peanuts, as determined by the proteomic spectral count approach [36]. When examining readily extractable PBS in raw peanut extracts by the proteomic intensity approach (integrated area under XIC curve), 89% were allergens, including PNA, while 11% belonged to the non-allergenic portion in the raw peanut extract [18].

Six of seventeen allergenic peanut groups (35%) are products of genes responsible for plant defence against any stress, including pathogenesis-related protein-10, Ara h 8 (PR-10, or Bet v 1 family member); Ara h 12 and Ara h13 (defensin family); Ara h 9 and Ara h 17, as nonspecific group 1 lipid transfer proteins (nsLTP-1, belonging to PR-14); and Ara h 16 (nsLTP-2, belonging to PR-14) (Table 1). PR-proteins are components of the innate immune system of plants and play an important role in the plant’s defence against pathogens [45]. Both PR-10 and PR-14 are small multifunctional proteins involved in plant response to abiotic and biotic stress conditions. Ara h 8 is a major peanut allergen in patients with combined birch pollen and peanut allergy [46]. Based on [18,36], we can say that these six minor allergen groups account for up to 12% of the relative abundance of the total allergenic load of peanuts in easily extractable fractions. All of these allergens are pan-allergens. Considering these data, the premise that food allergy is the fourth biological food quality control system (besides the established systems) that triggers allergic defence against food antigens associated with harmful substances, while exaggeration of this defence can lead to pathological food allergy [47], seems very logical.

### 2.2. Effects of Thermal Processing on Bioaccessibility and Allergenic Properties of Peanut Proteins

The functional properties of proteins are influenced by internal factors such as protein structure, conformation, amino acid composition, surface functional groups and hydrophobicity, and external factors such as pH, salts and solvents, ionic strength, temperature, pressure, shear stress and protein extraction and processing [48]. For example, peanut protein extraction expressed as mg per 100 mg of defatted flour was highest in various extraction conditions and methods of concentration assessment compared to nine tree nuts [49].

Food processing increases oxidative stress by introducing molecular oxygen (e.g., milling, blending and homogenising), removing natural antioxidants (e.g., physical and chemical separation of oil processing steps), destroying endogenous antioxidants (e.g., heat inactivation of antioxidant enzymes) and increasing pro-oxidant factors (e.g., light exposure producing singlet oxygen and release of protein-bound transition metals during heat treatments) [13]. Heating and fermentation are the most commonly used processing methods for food preparation [15].

**Figure 1:**
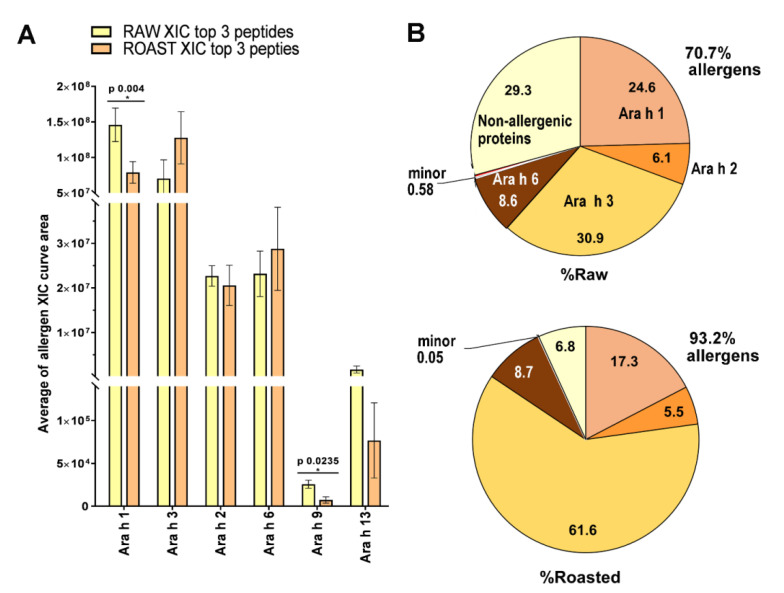
Raw and roasted PBS-based peanut extract protein profiles were calculated from Report S2, supplementary material [18]. (**A**) Label-free quantification of raw and roasted peanut allergens determined using PEAKS X Pro Studio (two groups, each with biological triplicates). Two tail, unpaired Student t-test was used to search for significant differences among XIC averages at *p* < 0.05. *—denotes significant difference in respect to raw peanut control sample. XIC—extracted ion chromatogram. (**B**) Relative abundance of allergens and non-allergenic proteins. The sum of the areas under the XIC curves for a given allergen was expressed as a percentage of the total (sum of all proteins identified in raw or roasted peanut extracts).

Boiling, roasting, frying and blanching are performed to improve peanuts’ nutritional value and flavour [6,50]. However, these processes affect peanut proteins’ bioaccessibility and extractability [18], ultimately affecting their digestibility [18], besides the antioxidant potential and allergenicity of the resulting extracts. The effect of roasting on the extractability of various peanut allergens and proteins and, thus, on their relative content can be easily understood by comparing readily extractable fractions from raw and roasted peanuts in Figure 1. Relative quantification of allergens showed that Ara h 1 and Ara h 9 are significantly higher in raw peanuts, whereas Ara h 3 and Ara h 6 are higher in roasted peanuts (Figure 1A), which is entirely consistent with other data [38,51]. Moreover, roasting resulted in a general enrichment of the major peanut allergens (23.2%) due to the double extent of Ara h 3, whereas the minor allergens practically disappeared in the roasted preparation (Figure 1B).

Roasting at 120 °C for 10 min reduces trypsin inhibitor activity by 42.5%; at 45 min, the reduction is almost complete (99.9% decrease in enzyme activity), while roasting at 120 °C for 150 min completely inactivates trypsin inhibitors [52]. This may be important in the use of raw, lightly roasted or briefly fried peanuts because such treatments may preserve the trypsin inhibitory potential of the allergens Ara h 1 [53], Ara h 2 [54] and Ara h 3 [55], which in turn may hinder protein digestion in the intestine. This is particularly relevant when the content of trypsin inhibitors (Ara h 1–3) is enriched, such as in roasted peanuts (Figure 1B), and their inhibiting activity could be partially preserved due to inefficient thermal treatment (duration and temperature of the roasting is dictated by nutritional, sensory and economic aspects).

Thermal processing of peanuts, such as dry roasting, frying and boiling, reduces their protein solubility [18,19,56,57]. The extraction efficiency of peanut proteins decreases with the increase in processing temperature, in that the protein yield of fried and dry-roasted peanuts decreases by 50–75% and 75–80%, respectively, compared to raw peanuts [58]. Furthermore, the roasting temperature influences the solubility of peanut proteins more than the duration of exposure to the respective roasting temperatures [58]. Schmitt et al., 2010, demonstrated that IgE binding increases in the insoluble peanut fractions with increased heating time, mainly due to the increase in the amount of insoluble proteins, whereas the overall protein solubility is reduced with processing [56].

For the extraction efficiency of peanut protein, the most important factor appears to be the pH of the extraction buffer used. Koppelman’s study observed the minimum protein extraction efficiency between pH 3 and 7 [59]. However, at lower pH (< 3), especially at pH < 2 corresponding to certain gastric conditions, and higher pH values (> 8) [59], especially in the pH range of 8–11, protein extraction became several times higher [58].

Molecular weight and quaternary structure certainly affect protein solubility. For example, Ara h 2 and Ara h 6 are small (15–20 kDa) monomeric proteins, while Ara h 1 and Ara h 3 are larger and form complexes up to 180–700 kDa and 360–380 kDa, respectively. In addition, Ara h 1 and Ara h 3 may aggregate during roasting and recombinant Ara h 1 during boiling [60], which limits their solubility, whereas Ara h 2 and Ara h 6 do not [59]. These remarkable differences in the size and quaternary organisation of peanut allergens may partly explain why Ara h 2 and Ara h 6 are more soluble than Ara h 1 and Ara h 3.

**Figure 2:**
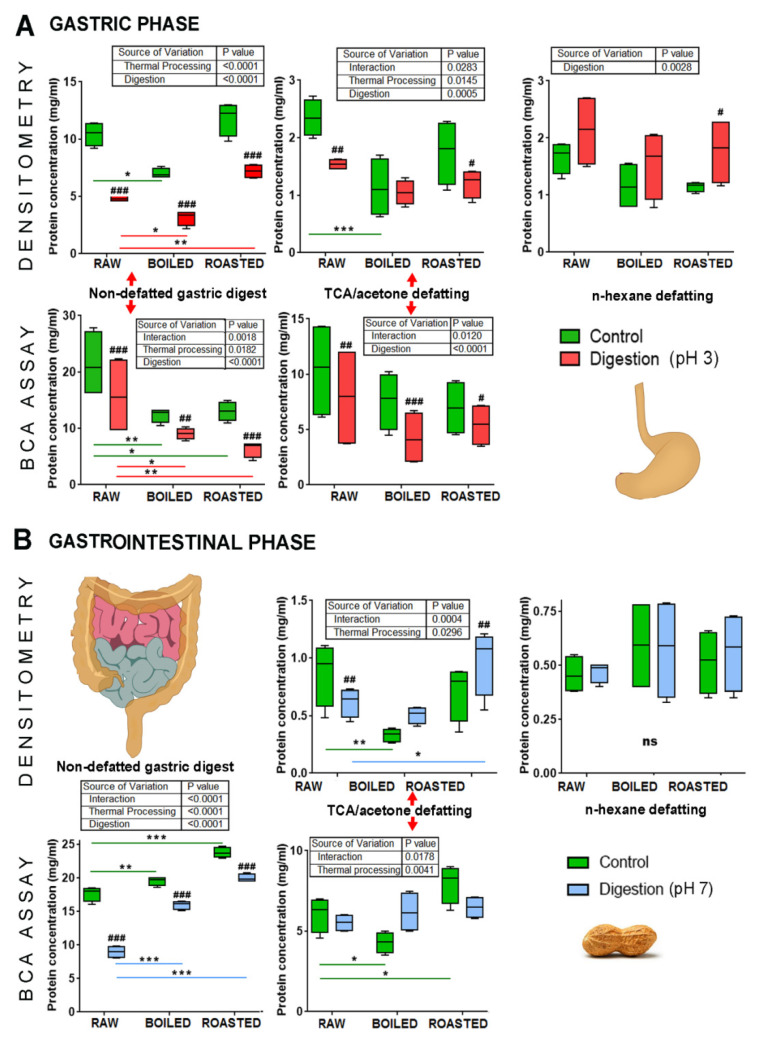
Averages of protein concentrations in different soluble phases of simulated peanut digestion. (**A**) Oral gastric phase protein content determined by BCA assay and densitometry tests, before and after defatting with n-hexane and trichloroacetic acid (TCA) precipitation. (**B**) Gastrointestinal phase. A two-way matched ANOVA was used on at least two separately performed digestions; ns, not significant; #, ## and ###, significant difference of digested sample compared to its control (*p* < 0.05, *p* < 0.005 and *p* < 0.0001, respectively). *, ** and *** denote significant difference of boiled or roasted peanuts in contrast to raw peanuts at *p* < 0.05, *p* < 0.005 and *p* < 0.0001, respectively. Adapted from Prodic et al., 2019 [19].

Although the study by Koppelman et al. (2018) was not conducted according to the INFOGEST protocol, it is appropriate to mention here that in the simulated oral phase (5 min, 37 °C with saliva-like buffer, pH 7.8) Ara h 2 and Ara h 6 are the first proteins extracted and exposed to a subject after consumption of lightly roasted peanuts, while Ara h 1 and 3 are not [59]. The previous paragraphs are related to the extractability in conditions not fully mimicking physiological conditions of digestion as it does INFOGEST protocol. Therefore, Figure 2, which was adapted from Prodic et al. [19], shows extracted protein concentrations from the raw, boiled and roasted peanut flours from the original, non-defatted state and defatted with *n*-hexane and trichloroacetic acid/acetone methods. In the acidic environment of the gastric phase, measured by the BCA test in non-defeated control samples of peanuts (green bars), the protein concentrations are 21.0 mg/mL for raw and 13.0 mg/mL for boiled and roasted peanuts. However, in an alkaline environment of the intestinal phase, the opposite trend was observed in thermally treated peanuts; raw peanut samples showed the lowest protein concentration and roasted peanuts the highest (Figure 2).

Low molecular weight peptides have been reported to have high solubility. Such peptides are more likely to achieve a physiological function than longer peptides because they are less susceptible to gastrointestinal hydrolysis. In addition, short peptides can be rapidly taken up by enterocytes and transported from the intestinal lumen into the bloodstream more efficiently than amino acids or intact proteins [61].

Many authors have demonstrated that thermal processing alters peanut allergenicity [62,63,64,65,66]. The current state of the art is that boiling [60] and frying lead to reducing peanut allergenicity [66], while the situation is somewhat ambiguous for roasted peanuts. However, more studies are pointing to the increased allergenicity as a consequence of roasting compared to the raw counterpart [63,67] than those who advocate a decrease [68] or no net effect [18,69]. Several mechanisms are responsible for the observed effects, such as structural modifications such as aggregation and degradation [64], chemical protein modifications from peanut roasting [18], etc. In summary, further and better-designed research is needed to explain such discrepancies.

### 2.3. The Effects of Thermal Processing on Bioaccessibility and Antioxidant Activity of Other Peanut Biomolecules

#### 2.3.1. Peanut Lipids and Lipid-Soluble Vitamins

Peanut oil consists of more than 95% neutral lipids (e.g., triglycerides and diglycerides) and a small part of polar lipids (phospholipids) and other bioactive substances such as tocopherols, sterols, squalene, etc. [70]. Lipids make up half of the weight of peanut kernels, and within the lipid content, unsaturated oleic (C18:1) and linoleic (C18:2) acids have the highest proportion, averaging 54% and 26%, respectively, followed by palmitic (C16:0) (10%) and behenic (C22:0) acids (3%) [22]. Therefore, peanut oil is 80% unsaturated with omega-9 (oleic acid) and omega-6 (linoleic acid) fatty acids [71]. From a nutritional point of view, high content of linoleic acid is desirable because it has a hypocholesterolemic effect [72]. Therefore, peanut varieties with high oleic acid content have been developed related to their excellent oxidative stability. In addition, the consumption of peanuts with high oleic acid content can help reduce disorders caused by oxidative stress [73].

Lipid oxidation occurs in food processing or storage due to heat, water, enzymes, oxygen, metal ions and light. The oxidation stability of lipids is one of the most important properties of oilseed processed products [74]. Furthermore, lipid-derived carbonyls are associated with rancid flavours and can interact with proteins to alter their functionality [74]. Therefore, it is vital to elucidate the potential relationship between the lipid structure and oxidative stability of peanuts, as it may affect the knowledge of the relationship between food allergies and the gut antioxidant environment, apart from the economic aspects related to the production, storage, selection and economic benefits of oilseeds and nuts.

Fatty acids and phospholipids are most affected by roasting; thermal degradation of phospholipids, oxidation of fatty acids and decomposition of oxidised fatty acids are the main features of roasting peanut fats [70]. Phospholipids are a large group of polar lipids in vegetable oils whose concentration increases markedly with moderate roasting but only slightly with intensive roasting. An increase in phospholipids indicates damage to the cell membrane. These results show that peanut oil’s di- and triglycerides exhibit perfect thermal stability during roasting [75]; the most favourable flavour and oxidation stability is achieved when the roasting temperature in the centre of the kernel is 185 °C [75]. After intensive roasting, palmitic, stearic, oleic and linoleic acid content decreases due to oxidative degradation [75]. The unsaturation of lipids is inversely related to oxidative stability [70]. All of these changes in colour, acid value, oxidative stability index, Maillard reaction and radical scavenging activity of peanut oil depend entirely on the degree of roasting. Dun et al. (2019) showed that roasting produces pyrazines, aldehydes and furans in peanut oil through the Maillard reaction between reducing sugars and amino acids, which exhibit fatty, nutty and roasted aromas [76].

Tocols, better known as vitamin E (lipid-soluble components consisting of tocopherols and tocotrienols)—natural antioxidants found in vegetable oils such as peanuts—include four isomers of tocopherols and four tocotrienols, designated α, β, γ and δ, respectively [77]. These antioxidants inhibit lipid peroxidation in foods by stabilising hydroperoxides and other free radicals, and peanuts are their richest source among nuts and legumes. The antioxidant activity decreases during each processing stage, as reported by Silva et al. (2010) [78]. Vitamin E is not degraded during blanching [79]. However, vitamins E and B are heat-sensitive and can be lost during roasting. In hazelnuts, roasting significantly reduced α, β and γ-tocopherols by 34%, 40% and 70%, respectively [6]. The extent of nutrient loss depends mainly on nut variety, roasting temperature and time.

#### 2.3.2. Peanut Carbohydrates

According to USDA nutritional data, peanut contains 16 g of carbohydrates, of which 9 g are dietary fibres (indigestible polysaccharides), and about 4 g are sugars (sucrose and reducing sugars), while the rest are starch. The American Diabetes Association classifies peanuts as a diabetes superfood because they have a low glycemic index and low loading and are high in fibre, calcium, potassium, magnesium, and vitamins A and E [24].

Digestible and soluble carbohydrates per se, such as sucrose, raffinose, and trehalose, have recently been associated with direct antioxidant properties [80], alongside studies showing synergistic effects when mixed with well-appreciated phenolic antioxidants such as gallic acid, catechin, and quercetin [81]. Furthermore, oxidation of endogenous soluble carbohydrates in Arabidopsis and barley suggests that higher sugars or even polysaccharides may act as a ROS scavenging mechanism in plants, ensuring cellular ROS homeostasis [80], and suggests that soluble sugars are part of an antioxidant system [82].

In addition to digestible carbohydrates, there are indigestible carbohydrates (which resist α-amylase and β-glucosidase in the oral phase and, to varying degrees, digestion in the rest of the digestive system). Most of them are better known as dietary fibre, partially degraded and fermented by the intestinal microbiota [83]. However, some have specific bioactive properties obtained from sources not associated with common foods (e.g., unusual mushrooms or sea cucumbers), which almost always benefit human health [84,85]. Discovered antioxidant property of herbal polysaccharides (dietary fibres) is now a hot spot in biomedicine research [86]. All these aspects are essential and relevant to our topic for two reasons: (1) these indigestible polysaccharides are important for regulating the homeostasis of the gut microbiota; their removal from the diet can lead to disruption of this homeostasis, which in turn causes inflammation and pathogenic gut conditions leading to the development of food allergies; (2) it has been shown that the antioxidant abilities of GIT are likely to be beneficial in the prevention/reduction of food allergies.

#### 2.3.3. Peanut Phenolics and Water-Soluble Vitamins

As established antioxidants, phenols protect plants from infections, tissue damage, and free radicals produced by photosynthesis and ultraviolet light [87]. Peanuts rank just behind walnuts in total phenolic content and third in antioxidant activity in the entire repertoire of tree nuts (TPC_peanut_ ~600 mg/100 g of fresh weight and 6 μmol TEAC/g) [88]. There is uneven saturation of phenolics compounds among peanut kernels and their skin, with the latter being substantially enriched in contrast to kernel [89,90,91]. Roasted peanuts have about 100 mg/100 g of total phenols, but the concentration increases six times when consumed with red skin [92]. Together with skin, roasted peanuts have a higher total phenolic content than known food sources, e.g., 10% more phenols per gram than blueberries, twice as many phenols as red wine, and 6 and 8 times more total phenols than green tea and cocoa drinks, respectively [92].

The main phenolics found in the peanut kernels are *p*-coumaric acid and its derivatives, while phenolic acids, flavonoids (catechins and procyanidins) [91] and stilbenes (resveratrol) were identified in the red skin of peanuts [90]. Thermal processing released *p*-coumaric acid at the expense of its derivatives, in descending order: oil roasting, dry roasting, and raw peanut kernels [89]. Although thermal processing altered the composition of antioxidants in the peanut kernel, total phenolic content and radical scavenging activity were preserved, while antioxidant capacity could increase depending on the peanut variety and harvest date [89].

Roasting reduces thiamine and carotenoids, especially in almonds and walnuts, but not in hazelnuts and pistachios [93]. Riboflavin cannot be destroyed by roasting peanuts [6]. However, it may be lost if exposed to air and light for a prolonged period. The effects of four thermal processing methods (boiling, autoclaving, microwaving and baking) on six antinutritive factors (trypsin inhibitor, α-amylase inhibitor, phytic acid, lectin and tannins) in peanuts showed that all heat treatments significantly reduced the antinutritive content and improved the in vitro digestibility of peanut proteins; however, roasting was most effective in reducing antinutritive substances and improving in vitro protein digestibility [94]. Nevertheless, not enough to reduce the allergenicity of Ara h 2 (trypsin inhibitor). Stilbenes are a class of phytoalexins and occur in response to infection, injury or stress [95]. The most crucial stilbene in peanuts is resveratrol, which reduces lipid peroxidation, oxidation and nitration of platelets and plasma proteins [96]. When peanuts are consumed with red skin, they provide about three times more resveratrol [24].

## 3. Digestibility of Biomolecules from Complete Food Matrix and Their Antioxidant Potential

Digestion can be described as the chemical breakdown of food by enzymes in the mouth, stomach and intestines with digestive juices, in which digested and absorbed nutrients are used for energy, growth and cell repair [97]. The GIT is divided into several parts, each necessary for proper digestion, depicting three phases: oral, gastric and intestinal, in which proteins are broken down into amino acids, fats are reduced into fatty acids and glycerol or carbohydrates are converted into simple sugar [97]. Besides macronutrient molecules serving as major energy and building blocks, digested food also includes secondary plant metabolites (e.g., polyphenols, terpenes) with different biological activities. Their activity and effective cellular absorption are related to their chemical structure under the given conditions [98] and to the effects of the food matrix in setting up the bioaccessibility of nutrients from the ingested meal. The matrix affects the fraction of a nutrient or compound released into the digestive fluid during passage through GIT [99].

In vitro digestion methods that simulate gastrointestinal (GI) conditions are increasingly used because they are rapid, safe and do not have the same ethical limitations as in vivo methods [100]. In vitro INFOGEST protocol is a static digestion method that uses a constant ratio of meal to digestion liquid and a constant pH for each step of the digestion process [101]. Food samples are subjected to consecutive oral, gastric and intestinal digestion, with a precise definition of parameters such as enzymes, electrolytes, bile, dilution, pH and digestion time based on available physiological data.

A precise and deep understanding of the oral–gastro–intestinal digestion process is mandatory if the aim is to fully comprehend the interplay between the allergenic potential and the antioxidant activity of the plant macro and micronutrients. Screening the antioxidant activity before and after digestion is warranted, ideally upon each digestion phase, as is done when bioaccessibility and antioxidant activity of polyphenolics is in focus [99]. Until now, significant research on the antioxidative properties of complex plant-based food matrices has been given to the secondary metabolites such as phenolics, polyphenolics and vitamins, as they are the most potent antioxidants, while the extent of polysaccharides and especially proteins have been neglected. Peanuts are an ideal model to simultaneously study various molecules’ contribution to antioxidant capacity since they are abundant in proteins (25–30%) and polysaccharides (22%), besides phenolics and vitamins. Protein and allergen digestion, as visualised in [18,19,35] and based on the data in Table 1, could release substantial antioxidative potential hidden in the plethora of peptides being liberated from the parental proteins that presumably had lower antioxidant capacity. In Table 2 are listed studies that have dealt with the antioxidative capacity of plant-based food with whole food matrix, being complex but rich in proteins (> 10% of raw plant material) or their components before and after the simulated gastrointestinal digestion.

Despite complex food antioxidant properties being investigated before and after digestion (comprised of proteins, carbohydrates, lipids and polyphenolics), none of the studies has addressed all relevant contributors. Therefore, observed effects are most likely not adequately explained if not misinterpreted. The studies in Table 2 showed the effect of digestion on the antioxidant activity of the complex food digests ascribing it to only phenolic compounds (five food sources) or solely to peptides (soybeans and oat). This effect of digestion on the antioxidant activity from different plants varies significantly depending on their chemical structure (Table 2). Thus, it is essential to determine the antioxidant contribution from understudied peptides in the studies that considered only phenolics (Table 2) and to consider the contribution of polyphenolics in the studies that explained the resulting antioxidant activity only by peptide involvement. Considering the simultaneously consumed foods in the daily diet, an in-depth evaluation of all biomolecules present in the investigated food is required to correctly explain total antioxidant capacity by Trolox standard (TEAC). Furthermore, it would be good to consider introducing new standards against which assays for expressing the antioxidative activity of peptides would be performed to secure reflection of the variety of antioxidative molecular species in complex food digests.

### 3.1. Perspectives on the Oral Phase of Simulated Digestion

The short oral digestion phase (~2 min) is important for carbohydrate-rich foods. Mixing of simulated saliva and administered food bolus is usually in a 1:1 ratio. Several factors are taken into account when designing the in vitro oral digestion step, such as various digestive enzymes (primarily lingual lipases and amylases), interactions with biopolymers in saliva and the bolus particle size reduction (250–1000 μm) to imitate chewing [101].

Besides carbohydrates being digested, hydrolysis of polyphenolic glycosides begins in the mouth through the action of β-glucosidase [108]. Furthermore, polyphenolics such as tannins have strong binding affinities with human basic salivary proteins rich in proline and histidine and form stable non-covalent and covalent associations, thus supporting the premise that these salivary proteins act as a defence against tannins [109].

The physiological effect of dietary fibres is mainly related to their behaviour during gastrointestinal digestion [83]. Therefore, it seems that antioxidant non-digestible polysaccharides might be partially digestible in our GIT. However, the mechanisms of this partial digestion and outcomes of the resulting antioxidant capacity of the digests compared to the starting material remain to be fully resolved and explained [110,111]. It is undoubtedly intriguing to reveal if antioxidant polysaccharides can behave similarly to proteins that possess some antioxidative capacity while in the intact form, which is being multiplied and enlarged upon their digestion and release of antioxidant peptides (Table 2). Designing studies to decipher the change of antioxidative properties before and after digestion would greatly aid in the fundamental understanding of food digestion and food allergy and in addition to designing more impactful foods as nutraceuticals.

Even though proteins are not digested in the mouth, small proteins (e.g., less than 20 kDa) can be liberated and become bioaccessible in the pH environment of the oral cavity and saliva (pH 6,5–8,5) [59]. Ara h2 and Ara h6 are the first allergens an individual is exposed to upon ingesting peanut-containing food and are quickly bio-accessible in the mouth, potentially explaining their extraordinary allergenicity [59].

### 3.2. Perspectives on the Gastric Phase of Simulated Digestion

A gastric phase per se is important as a tool for evaluating the stability or digestion resistance of proteins and allergens. For example, there is a correlation between resistance to pepsin digestion and allergenic potential, a relatively moderate predictor of allergenicity. Still, gastric digestion is used to monitor the digestibility and, based on these results, to predict the allergenicity of the novel and recombinantly produced proteins [112].

Digestion of proteins and lipids starts in the acidic conditions of the gastric phase under the influence of pepsin and the action of gastric lipase [101]. Surprisingly, gastric lipase is usually omitted in most in vitro static digestion research [101], which is not a proper solution, especially if the food matrix has lipids above 10% [113]. In addition, most dietary lipids are present in emulsified droplets, and it is hypothesised that gastric lipolysis could help increase emulsification in the stomach, thus improving the bioaccessibility of lipophilic phytochemicals [97]. Thus, INFOGEST 2.0 recommends adding gastric lipase to the gastric digestion phase [101]. However, there are many modifications of the INFOGEST protocol in the literature published, whose reasons for modifications are not justifiable [21] since INFOGEST aims to secure results comparability between different digestion studies [101]. In addition to it, the INFOGEST protocol recommends assessing the whole food matrix digestion, and Di Stasio et al. (2017) and Prodic et al. (2019) expressed concern about the relevance of digestion results of pure proteins or their defatted extracts due to the lack of food matrix interactions and altered bioaccessibility of protein determinants [19,114]. A study by Prodic et al. (2019) showed that the different defatting procedures applied after simulated digestion affects protein extraction and digestion profiles [19] and have developed a method for a successful isoelectrofocusing step with the inclusion of the complete, undefeated samples [115], including the undefeated gastric digestion of thermally processed hazelnuts [113].

The digestion of whole raw and thermally processed peanuts using the INFOGEST protocol, mimicking physiologically relevant conditions, shed a new light [19,35]. Digestion-resistant forms of Ara h 1 and 3 were found to be stable under gastric digestion conditions for 120 min in the presence of the food matrix [35], with an estimation that Ara h 1 and Ara h 3 are 500 times less digestible by pepsin in whole peanut kernel than in the study of Koppelman et al., 2010, done with pure protein extracts [116]. In addition, it is renowned that Ara h 2 and Ara h 6 are resistant to pepsin, trypsin and chymotrypsin digestion in various protocols, producing large stable fragments with preserved immunological potential [117]. The same stands when INFOGEST was applied on the whole peanut, Ara h 2/6 were poorly digested even after 120 min of exposure to pepsin in heat-treated peanut samples [19]. Low molecular weight proteins (<10 kDa) and digestive fragments were resistant to pepsin digestion [35].

A reasonable explanation for the reduced digestibility of peanut protein may be the “masking effect” of the matrix, delaying or hindering protein degradation and changing the pattern of peptide fragments released by proteolysis. Protein-rich matrices saturate pepsin, allowing sensitive proteins to escape digestion for long periods. In addition, peanuts contain high protein content, higher lipids and a moderate extent of polysaccharides, which can affect proteolysis, sparing several immunologically active polypeptides. Some polysaccharides have been shown to reduce the digestibility of peanut allergens [118]. Furthermore, proteolytic enzymes could play a role in the bioaccessibility of polyphenols after releasing them from the binding interactions with dietary proteins, as observed in the gastric phase after pepsin action [97]. In addition, acidic pH during the gastric phase has been shown to protect polyphenols from degradation [119].

### 3.3. Perspectives on the Intestinal Phase of Simulated Digestion

Pancreatic enzymes, including proteases (trypsin and chymotrypsin), amylases and lipases, work together with all digestive enzymes (maltase, lactase, α-dextrinase and peptidases) to break down food. Therefore, limiting oxygen levels, including sufficient bile salt concentration, α-glucosidase activities and the presence of lipolytic, amylolytic and proteolytic enzymes to digest specific nutrients, are critical for optimal phytochemical release [101]. In addition, some data support the role of phenolic compounds as inhibitors of intestinal lipase and trypsin, which can be viewed in an antinutrients light [120], but also could affect the antioxidative potential by reducing protein digestibility and preventing the liberation of antioxidative peptides.

The amount of proteins and peptides that reaches the intestinal system depends on the abundance of protein in a particular food and its resistance to pepsin digestion. For example, Ara h 2 and Ara h 6 remain stable after 120 min of digestion due to their tightly coiled structure that resists heat and gastrointestinal digestion [117]. In addition, they possess trypsin inhibitor activities [54]. This tryptic-inhibiting activity greatly contributes to the reduced digestibility of peanut proteins and peptides in the intestinal phase, protecting the rest of the proteins against tryptic digestion [19]. Ara h 3 isoforms were even identified in raw and roasted peanuts after 120 min of gastrointestinal digestion [19,116]. However, in the study by Rao and colleagues, boiled peanut proteins are degraded faster than roasted peanuts [65] because the proteins remain trapped in their matrix due to aggregation during the roasting. Therefore, collecting as much data as possible on protein structural changes is necessary to understand their influence on allergenicity and bioaccessibility during digestion.

Although many papers, such as Dyer et al., 2018 [121], speculate about the possible contribution of post-translational and chemical modifications (PTMs) towards affecting allergenicity, no one studied them extensively by quantitative profiling. The partial exception to this is our recent study that addresses readily extractable allergenic portion from the defatted raw and roasted peanut flours with unspecified PTMs profiling included, which were confirmed in part by commercially available antibodies directed against specific modifications [18]; however, the data were not obtained from natural or simulated digestion processes. Similar to this, there were studies dealing with pure peanut extracts, not involving the simulated digestion process, revealing glycosylation of Ara h 1 [122], hydroxyproline on Ara h 2 [123] and on Ara h 1 and Ara h 3 [124] and those created by roasting, such as advanced glycation end products, carboxymethyl lysine on Ara h 1 and Ara h 3 [18]. The Maillard reaction is commonly considered one of the reasons for increased sensitisation to fried peanuts [125]. Perhaps those voluminous modifications imposed by the Maillard reaction during the roasting can interfere with and impede digestion, as in the case of glycated ovalbumin, where trypsin could not hydrolyse glycated Arg/Lys cleavage sites [126].

Even though peanut is one of the most important industrial legumes, popular in the human diet and established as a functional food, there are no studies on the antioxidant properties of peanut in the gastrointestinal digestive phases, as previously suggested (to first study the proximal content and antioxidant properties and follow them according to the main digestive phases).

## 4. Food-Derived Bioactive Peptides with Antioxidant Activity

The antioxidant activity of protein-derived peptides was one of the first to be revealed among fifty and more bioactivities [127], such as antibacterial, antifungal, antiviral, antihypertensive (via ACE-2 inhibition), anti-hyperglycaemic, anti-cancer as lunasin from soybean [128] or antiaging properties [9]. Strictly, bioactive peptides are referred to as those released from the parent protein by enzymatic and chemical cleavage, in contrast to the peptides being directly synthesised [129]. However, the most attractive feature of peptides is their ability to show very few side effects in humans due to their natural sources.

Food-derived bioactive peptides can be released during in vivo digestion, in vitro enzymatic hydrolysis or food processing, including fermentation, germination and ripening [130]. Producing peptides using hydrolytic enzymes seems to be the most promising technique for creating more potent antioxidants since peptides have a significantly higher antioxidant potential than intact proteins [131]. For example, reducing power, DPPH radical scavenging and metal ions chelating activities increased gradually with the increase in the hydrolysis time and the concentration of enzymes used (*Aspergillus* protease, trypsin, pepsin and papain) for hydrolysis of goat skimmed milk proteins [132], and of rapeseed proteins with *Bacillus subtilis* [133]. The increased ability of protein hydrolysates to reduce free radical reactivity is associated with increased solvent exposure of amino acid residues involved in free radical scavenging, as well as the ability of peptides to reduce the energy of released free radicals, which reduces its ability to oxidise lipids [131].

Peptides below 3000 Da have been reported to possess the highest antioxidant power (2–20 amino acids) [130]. The chemical structures of the peptides are the main factor influencing their antioxidant activity, which is usually checked by the in vitro chemical and cellular antioxidant assays. Hydrophobic amino acid residues, such as His, Trp, Phe, Pro, Gly, Lys, Ile and Val, are attributed to strong radical scavenging activity in oxidative reactions, especially for enzyme-catalysed reactions, due to the imidazole ring from histidine as a vital proton donor [134]. In addition, the indole and pyrrolidine rings in Trp and Pro and Tyr, Cys and Met can also serve as hydrogen donors through their hydroxyl and thiol groups, thus acting as scavengers of hydroxyl and other free radicals [134,135]. Peptides obtained by simulated gastrointestinal digestion of hemp seeds, WVYY and PSLPA, showed the best antioxidant and antihypertensive properties, pointing to the presence of three aromatic amino acids, Trp, Tyr and Pro, in acquiring the pronounced antioxidative peptide properties [136].

Data regarding the antioxidant peptides derived from the nuts are emerging [135,137,138,139,140]. The type and position of amino acids, grand average of hydropathicity index and its mass are smartly used to select six candidate peptides (upon analysis of BIOPEP database): FSEY, QIESW, SEGFEW, IDLGTTY, GEGFFEM and NLNQCQRYM from hazelnut protein hydrolysates with higher antioxidant capacity than glutathione against linoleic acid oxidation [135]. The peptides with C-terminal Tyr prevented linoleic acid oxidation better than others [135]. In addition, the same research group synthesised various dipeptides with antioxidant-active and inactive amino acids and demonstrated that dipeptides possessing cysteine residue showed a striking ability to scavenge O2•− [135]. While Cys as the sole amino acid was excellent in inhibiting activity against linoleic acid oxidation (better than glutathione), dipeptides with Cys residue did not have more activity against linoleic acid oxidation than dipeptides with Met, Trp and Tyr [135].

Concerning peanuts, Chen et al., 2007, reported that peanut protein hydrolysates produced by commercial alcalase could inhibit linoleic acid peroxidation and scavenge DPPH radicals [137]. Hwang et al., 2008, found that roasted and defatted peanut esterase hydrolysate could moderate linoleic acid autoxidation, scavenge DPPH free radicals and inhibit human LDL oxidation in vitro [138], attributing these effects to Maillard reaction products from the lipid–carbohydrate–protein system in roasted peanuts. Zhao et al., 2022 reviewed all antioxidant peptides from nuts generated by various enzymatic hydrolyses since 2010, and among 33 references compiled, 10 were dedicated to peanut preparations [140]. Only one study employed pepsin as the representative of GIT proteases, showing that peanut peptides obtained by pepsin possess one-third lower antioxidant activity by DPPH scavenging radical test than the hydrolysates obtained by alcalase and that this activity does not change concerning the varying degree of hydrolysis [141]. Therefore, we can conclude that peanuts represent an excellent source of antioxidative peptides.

## 5. Conclusions and Future Prospects

Today, an increasing number of people are paying attention to a healthy lifestyle—of which adequate food intake is crucial. Peanuts have plenty of lipids, proteins and carbohydrates and are considered one of the best functional plant foods in terms of versatility and coverage of the average daily human nutritional requirements. At the same time, peanuts are one of the most allergenic foods, with 75–85% of the total protein content being allergens in raw versions, while roasting additionally increases allergenic share above 93%. What is happening in the GIT, with the digestion of complex food, in terms of paralleled allergen destiny and antioxidative potency of the whole food, still requires mechanistic and complete answers. However, the lack of strict compliance with in vitro digestion protocols mimicking physiological conditions, and post-digestion defatting procedures, prevents the generation of physiologically relevant results and the possibility for their inter-laboratory comparison regarding gastrointestinal stability/resistance of peanut allergens. Therefore, future directions for research should include measuring and considering the antioxidative contribution of proteins and polysaccharides upon digestion in the complex, protein-rich food matrices by designing needed proteinaceous and carbohydrate digestion control experiments. Our premise that proteins are the neglected source of antioxidant potential is based on the property of a single protein macromolecule to release several peptides and a bunch of amino acids with several times higher total antioxidant capacity. In addition to this qualitative aspect, this review offered a tool for the quantitative contribution assessment of the peanut proteins in the GIT antioxidant milieu by providing the relative extent of peanut proteins and allergens in the proximal and gastric digestion phases (Table 1). The same can be applied to the dietary fibres partially digestible by human microbiota. In addition, the future direction in researching the relationship between allergenicity and antioxidant food capacity should be based on the whole food or its preparations, considering all relevant macromolecules and those small molecules already renowned for their antioxidant properties.

It is known that trypsin is not the protease of choice when considering food protein hydrolysate production with enhanced antioxidant activities since peptides will end with K or R amino acids at their C terminus, which is regarded as non-active amino acids concerning antioxidant capabilities. However, the behaviour of all plant-based hydrolysates, especially those obtained by enzymes present in the GIT, at which point hydrolysis is an emerging increase of antioxidant capabilities, supports our premise that the contribution of proteins in antioxidant properties of plant foods rich in proteins is underestimated. In addition, studies that aimed to characterise novel bioactive peptides with antioxidant activity from hydrolysates could be used to assess the extent of protein share in resulting antioxidant capacity during the digestion of complex plant food matrices with substantial protein share (higher than 10%). Moreover, we should not forget that microbiota’s proteolytic machinery that is more versatile than commercial microbial enzymes used for hydrolysate preparations, which is important when considering the final digestion peptide portfolio and its antioxidant potential.

Until the mechanistic details of primary sensitisation are fully revealed, including the involvement of molecules stimulating innate immunity and all antigens, we cannot answer why peanut allergy is more severe than soy allergy. In summary, the review presents the importance of deciphering the actual antioxidant contribution of proteins and polysaccharides in peanuts (besides its polyphenolics and vitamins) upon gastrointestinal digestion, taking into account the proximal composition, bioaccessibility, antioxidant activity and immune properties of allergenic proteins/peptides obtained during digestion. This knowledge will help establish a fuller scope of connections between food allergy and antioxidant activity.

## Figures and Tables

**Table 1:** Allergens and non-allergenic proteins identified and relatively quantified by proteomics approach in mature peanut seed (*Arachys hypogaea*).

Protein Family (Pfam Database)	Allergome/Enzyme	UniProt Access.	Protein Name from UniProt Database	Relative Abundance % (XIC Area)			
				Avrg. Mass (kDa)	Gastric Ctrl [19]	Gastric Digest [19]	PBS pH7,4 [18]
1. Cupin (Vicillin-type, 7S globulin)	Ara h 1	Q6PSU4	Conarachin (Fragment)	48.1	0.1	0.3	/
		P43238 = N1NG13	Seed storage protein Ara h1	71.3	0.4	10.4	/
		Q6PSU3	Conarachin (Fragment)	66.6	0.3	2.4	/
		P43237 = B3IXL2	Allergen Ara h 1 clone P17	70.3	0	4.9	20.3
		E9LFE7	7S conarachin (Fragment)	15.9	0	0	/
		Q6PSU5	Conarachin (Fragment)	33.6	0	0.1	/
		Q6PSU6	Conarachin (Fragment)	34.1	0	0.1	/
		E5G076	Ara h 1 allergen	70.8	0.3	0	4.3
2. Conglutin (2S albumin)	Ara h 2	Q6PSU2	Conglutin-7	20.1	/	/	6.1
	Ara h 6	Q647G9	Conglutin	16.9	/	/	8.6
	Ara h 7	B4XID4	Ara h 7 allergen	19.3	/	/	0
		Q647G8	2S protein 2 (Ara h 7.0301)	18.5	/	/	0.0
3. Cupin (Legumin-type, 11S globulin, Glycinin)	Ara h 3	A1DZF0	Arachin 6	60.4	1.7	7.7	0
		Q9FZ11	Gly1	60.4	1.3	0.2	0.3
		B5TYU1	Arachin Arah3 isoform	60.6	3.1	6.6	/
		Q5I6T2	Arachin Ahy-4	60.7	0.8	0.1	0
		Q647H4	Arachin Ahy-1	61.5	0.9	0.1	0.3
		Q647H3	Arachin Ahy-2	61.5	17.6	3.3	4.4
		Q8LKN1	Allergen Arah3/Arah4	61.7	1.6	4.1	/
		Q9SQH7	Glycinin	61.0	0.8	5.5	0
		Q8LL03	Trypsin inhibitor (Fragment)	25.5	0.8	0	/
		Q6T2T4	Storage protein	61.5	0	0.1	/
		Q6IWG5 = Q0GM57	Glycinin (Fragment)	58.1	62.9	49.3	24.9
		E5G077	Ara h 3 allergen	58.3	0.9	4.3	/
		Q647H2	Arachin Ahy-3	54.6	2.2	0	1.0
	/	E9LFE9	11S arachin	28.3	0	0	1.4
		E9LFE8	11S arachin (Fragment)	28.3	0	0	8.8
		A1E2B0	11S seed storage globulin B1	33.5	0	0	4.4
		A1E2B1	11S seed storage globulin B2	31.3	/	/	1.6
		A7LIS5	Germin-like protein	23.5	/	/	0
		D4NXQ0	Germin-like protein subfamily 3 member 3	23.4	/	/	0
		Q647H1	Conarachin	75.9	/	/	0.4
4. Profilin	Ara h 5	D3K177	Profilin	14.2	Johnson et al., 2016 [38]		
5. Pathogenesis-related protein, PR-10, Bet v 1 family member	Ara h 8	Q6VT83	Ara h 8 allergen	17.0	/	/	0
		B1PYZ4	Ara h 8 allergen isoform 3	16.9	/	/	0
		B0YIU5	Ara h 8 allergen isoform	16.4	/	/	0
		Q2YHR1	Pathogenesis-related protein 10 (Fragment)	14.4	/	/	0.1
		B2ZGS2	Pathogenesis-related class 10 protein	16.9	/	/	0.1
6. Plant LTP family 1	Ara h 9	B6CEX8	Non-specific lipid-transfer protein	11.7	/	/	0.2
		B6CG41	Non-specific lipid-transfer protein (Fragment)	9.1	/	/	0.2
	Ara h 17	A0A510A9S3	Non-specific lipid-transfer protein 1	9.4	http://allergen.org/viewallergen.php?aid=831, accessed on 1 February 2023		
7. Plant LTP family 2	Ara h 16	A0A509ZX51	Non-specific lipid transfer protein 2	7.0	http://allergen.org/viewallergen.php?aid=830, accessed on 1 February 2023		
8. Oleosin family	Ara h 10	Q647G5	Oleosin Ara h 10.0101	17.7	/	/	0.1
		Q647G4	Oleosin 17.8 (Ara h 10.0102)	15.5	/	/	0.1
	Ara h 11	Q45W86	Oleosin Ara h 11.0102	14.3			
		Q45W87	Oleosin Ara h 11.0101	14.3			
	Ara h 14	Q6J1J8	Oleosin Ara h 14.0103	18.4			
		Q9AXI0	Oleosin Ara h 14.0102	18.5			
		Q9AXI1	Oleosin Ara h 14.0101	18.4			
	Ara h 15	Q647G3	Oleosin Ara h 15.0101	16.9	[39]		
9. Defensin family	Ara h 12	B3EWP3	Defensin 1	7.9	[40]		
	Ara h 13	C0HJZ1	Defensin 3	8.3	/	/	0.2
10. Cyclophilin family	Ara h 18		- peptidyl-prolyl cis-trans isomerase				
11. Leguminous lectin family	Ara h agglutinin	P02872	Galactose-Binding Lectin	29.3			
		Q38711	Galactose-binding lectin (Fragment)	29.1	0	0	0.3
		A0A089ZXL7	Peanut agglutinin variant	29.4	0	0	0.1
		P02872	Galactose-binding lectin	29.3	2.5	0	/
		Q43373	Galactose-binding lectin	29.6	0	0	/
12. Chitinase class I	Hydrolase	Q42515	Chitinase (Class II)	28.9	/	/	0.1
13. Proteasome subunit		Q1PCR5	20S proteasome beta subunit Endopeptidase	24.0	/	/	0
		N1NJN8	Proteasome subunit Peptidase T1B	23.5	/	/	0
		B4UWD5	Proteasome subunit alpha type Peptidase T1A	15.0	/	/	0.1
14. Phospholipase D family		Q2HWT8	Phospholipase D	92.3			
15. RuBisCO large chain family	Lyase	O20356	Ribulose bisphosphate carboxylase	51.9			
		A0A191UJ50	Ribulose bisphosphate carboxylase large chain	52.6	/	/	0.1
16. Glyoxalase/ /Dioxygenase superfamily		B4UWB4	Lactoylglutathione lyase	23.1			
17. Fructose-bisphosphate aldolase class-I		T2B9M0	Fructose-bisphosphate aldolase	38.4	0.1	0.3	2.7
18. PEPCase type 1 family		C9W981	Phosphoenolpyruvate carboxylase	116.4	0	0	/
19. Short chain dehydrogenase	Oxidoreductase	A7LB60	Steroleosin-A	38.8			
20. Glutathione peroxidase family		B4UW79	Glutathione peroxidase	24.4			
21. Redoxin		Q06H32	Glutaredoxin-dependent peroxiredoxin	17.5			
22. Glyceraldehyde 3-phosphate dehydrogenase C		A0A0A6ZDP1	Glyceraldehyde-3-phosphate dehydrogenase C2	20.1	0	0	4.8
		A0A0A6ZDT0	Glyceraldehyde-3-phosphate dehydrogenase C2	20.1	0.3	0	0.2
23. Lipoxygenase family		Q4JME6	Lipoxygenase	97.5	0	0	/
		Q4JME7	Lipoxygenase	97.6	0	0	/
		Q9M5D3	Lipoxygenase	97.6	0.5	0	/
24. SDR family		E6Y9A9	Enoyl-ACP reductase	41.5	/	/	0
		D8KXZ8	Enoyl-ACP reductase 1–3	41.5	/	/	0
25. Alcohol dehydrogenase		H6U596	Alcohol dehydrogenase (Fragment)	19.4	/	/	1.0
26. NAD dependent epimerase/dehydratase family		B4UW57	Putative dihydroflavonol reductase (Fragment)	24.3	/	/	0
27. Cu-Zn superoxide dismutase family		Q1HDS7	Superoxide dismutase [Cu-Zn]	15.2	/	/	0
		Q45W82	Cu-Zn superoxide dismutase	15.1	/	/	0
28. Glutathione S-transferase. N	Transferase	B4UW81	Glutathione S-Transferase 2	20.3			
29. Beta-ketoacyl-ACP synthases family		E6Y9A7	Beta-ketoacyl-ACP synthetase I	49.9	[38]		
30. UDPGP type 1 family		Q06H19	UDP-Glucose Pyrophosphorylase	16.9			
31. Leucine Rich Repeat family		A0A290G0J9	Resistance protein (Fragment)	57.8	0.2	0	/
		A0A290FZZ3	Resistance protein (Fragment)	41.5	/	/	0
32. Protein kinase family		A0A290GKJ7	Resistance protein (Fragment)	54.6	0.1	0	/
33. RNA polymerase Rpb1		A0A191UJ63	DNA-directed RNA polymerase subunit beta	157.0	/	/	0
34. SHMT family		A0A0A6ZDR9	Serine hydroxymethyltransferase	52.0	/	/	
		A0A0A6ZDT3	Serine hydroxymethyltransferase	52.0	/	/	
35. Thiolase family		A0A0R4UXQ1	3-ketoacyl-CoA thiolase	48.3	/	/	0.1
36. NDK family		Q45W80	Nucleoside diphosphate kinase	16.4	/	/	
37. Glutathione S-transferase. N-terminal domain		B4UW81	Glutathione S-transferase 2	20.3	/	/	0.6
38. AMP-binding enzyme	Ligase	A0A109QJM5	Long chain acyl-CoA synthetase 4	74.3	0	0	/
39. Complex1_ 49kDa 1	Translocase	A0A191UJC0	NAD(P)H-quinone oxidoreductase subunit H. chloroplastic	145.5	0	0	/
40. Chalcone isomerase family	Isomerase	J9QGM3	Chalcone-flavonone isomerase family protein	27.6	0	0	/
41. LEA type 1 family	/	Q2PXN4	Seed maturation protein LEA 4	5.8			
42. LEA type 2 family	/	Q2PXN9	Dessication Protectant Protein LEA 14	16.2			
43. LEAP-3	/	E5FHY6	Late embryogenesis abundant protein group 3 protein (Fragment)	18.0	/	/	0
44. LEA type seed maturation protein family	/	E5FHZ2	Late embryogenesis abundant protein group 5	27.4	/	/	0.1
	/	N1NKG9	Late embryogenesis abundant protein	26.0	/	/	0.2
45. Small hydrophilic plant seed protein family	/	Q4U4M1	LEA protein	10.4			
		E5FHY2	Late embryogenesis abundant protein group 1 protein	10.7	/	/	0
		E5FHY1	Late embryogenesis abundant protein group 1 protein	10.1	/	/	0
46. EF-hand domain pair	/	Q6PWX0	Calmodulin	16.7			
47. Glutathione S-transferase, C terminus	/	Q1PCR4	Putative IN2-1 Protein	16.1			
48. Cystatin family	/	B3GR01	Cysteine proteinase inhibitor	10.7			
49. Bowman-Birk serine protease inhibitor family	/	Q0PKR5	Proteinase inhibitor	7.9			
	/	Q2VMU0	Serine protease inhibitor	12.2			
	/	Q7X973	Bowman-Birk trypsin inhibitor	9.5			
50. Plant PEC family metalothionein	/	Q0Q0Q8	Type 4 metallothionein	9.1			
51. NB-ARC family	/	Q2KQ45	Resistance protein PLTR (Fragment)	19.6	0.5	0	/
	/	A0A1B3TNS1	NBS-LRR type disease resistance protein	108.6	0	0	/
52. DnaJ family	/	A0A126DIH0	DnaJ	39.1	0	0	/
53. Annexin family	/	A0A0F7GF62	Annexin	36.1	/	/	0.1
		A0A0F6VX63	Annexin	36.1	/	/	2.3
		B4UW70	Fiber annexin	18.8			
54. Phosphatidylethanolamine-binding protein family	/	K7PQ65	Mother of FT and TFL1	19.2	/	/	0
55. TCTP family	/	Q06H31	Translationally controlled tumour-like protein	19.1	/	/	0.1
56. Actin-binding proteins ADF family	/	Q2PK12	Actin depolymerising factor-like protein	16.1	/	/	0
57. Trypsin and protease inhibitor	/	B4UWB2	Kunitz trypsin inhibitor 4	22.1			
58. Protein of unknown function	/	B4UWA3	Putative Uncharacterised Protein	20.2			
59. Endoribonuclease L-PSP	/	B4UWE2	Perchloric Acid Soluble Translation Inhibitor Protein	20.3			
60. Major intrinsic protein	/	Q06H34	Tonoplast intrinsic protein alpha TIP	19.2			
61. Ubiquitin family	/	Q06H40	Ubiquitin-Ribosomal Protein S27a	21.4			
62. Actin family	/	A0A0A1EUV7	Actin (fragment)	35.9	[38]		

**Table 2:** Overview of studies assessing the potential change in the antioxidative capacity of protein-rich, plant food sources before and during the simulated gastric/gastrointestinal digestion.

Reference	Food/Biomolecules as the Contributor of Antioxidant Capacity	Simulated Digestion Protocol	Antioxidative Test Employed	Outcome
Coelho et al., 2021 [21]	Tomato pomace/phenolics	Modified INFOGEST 2.0 [102]	ABTS·+ (TEAC)	Significantly increased in GP (more) and IP (less)
Comert and Gokmen, 2022 [20]	Hazelnuts/phenolics Flaxseed/phenolics	INFOGEST 1.0	ABTS·+ (TEAC)	Significantly increased in GP (more) and IP (more)
Tome-Sachez et al., 2021 [103]	Germinated whole-wheat grain/phenolics	INFOGEST 2.0	DPPH, ORAC, ABTS·+, FRAP	Increased in GP (more) and IP (significantly compared to GP)
Tonolo et al., 2020 [104]	Soy/peptides	INFOGEST 1.0	ABTS·+, DPPH	Decreased after GP and IP
Calvo-Lerma et al., 2020 [105]	Chia seed/phenolics	INFOGEST 1.0	DPPH	Decreased after GP and IP
Darewicz et al., 2022 [106]	Oat/peptides	INFOGEST 1.0	DPPH	Increased in GP (more) and IP (significantly compared to GP)
Sánchez-Velázquez et al., 2021 [107]	Oat/peptides	INFOGEST 2.0	ABTS·+, DPPH	Increased in GP (more) and IP (significantly compared to GP)

## Data Availability

Data is contained within the article.
